# RNA-Binding Motif Protein 3 as a Therapeutic and Prognostic Target for Drug Discovery

**DOI:** 10.3390/cimb48080815

**Published:** 2026-08-12

**Authors:** Marvin A. Larbi, Robert Getzenberg, Dmitriy Minond

**Affiliations:** 1 Department of Pharmaceutical Sciences, Rumbaugh Goodwin Institute for Cancer Research, Barry and Judy Silverman College of Pharmacy, Nova Southeastern University, 3321 College Avenue, Fort Lauderdale, FL 33314, USA; ml2900@mynsu.nova.edu; 2 Astellas Pharma Global Development, 2375 Waterview Dr, Northbrook, IL 60062, USA; rhgetzenberg@gmail.com

**Keywords:** RNA binding motif 3 (RBM3), therapeutic target, cancer biomarkers, cell stress response, prognostic indicators

## Abstract

This comprehensive literature review delves into the multifaceted roles of RNA-binding motif protein 3 (RBM3) in cellular processes, disease pathogenesis, and therapeutic potential. RBM3 has been implicated in shaping cell morphology, synaptic protection in neurodegenerative conditions, and regulating gene expression through binding to specific RNA sequences. In cancer, RBM3 exhibits contrasting effects, influencing cell proliferation, tumorigenic potential, and RNA splicing. Clinical studies suggest RBM3 as a predictive biomarker in chemotherapy response for muscle-invasive bladder cancer. Despite promising therapeutic implications in neuroprotection and cancer, challenges persist in understanding the regulatory mechanisms and clinical behavior of RBM3. Further research is warranted to elucidate the molecular mechanisms underlying RBM3’s diverse functions and its significance as a potential target for personalized medicine in cancer therapy. This review underscores the pivotal role of RBPs, particularly RBM3, in disease progression and highlights the need for continued investigation to harness their therapeutic potential effectively. This review evaluates evidence available through December 2025, with particular emphasis on studies published between 2010 and 2025.

## 1. Introduction

**Figure 1:**
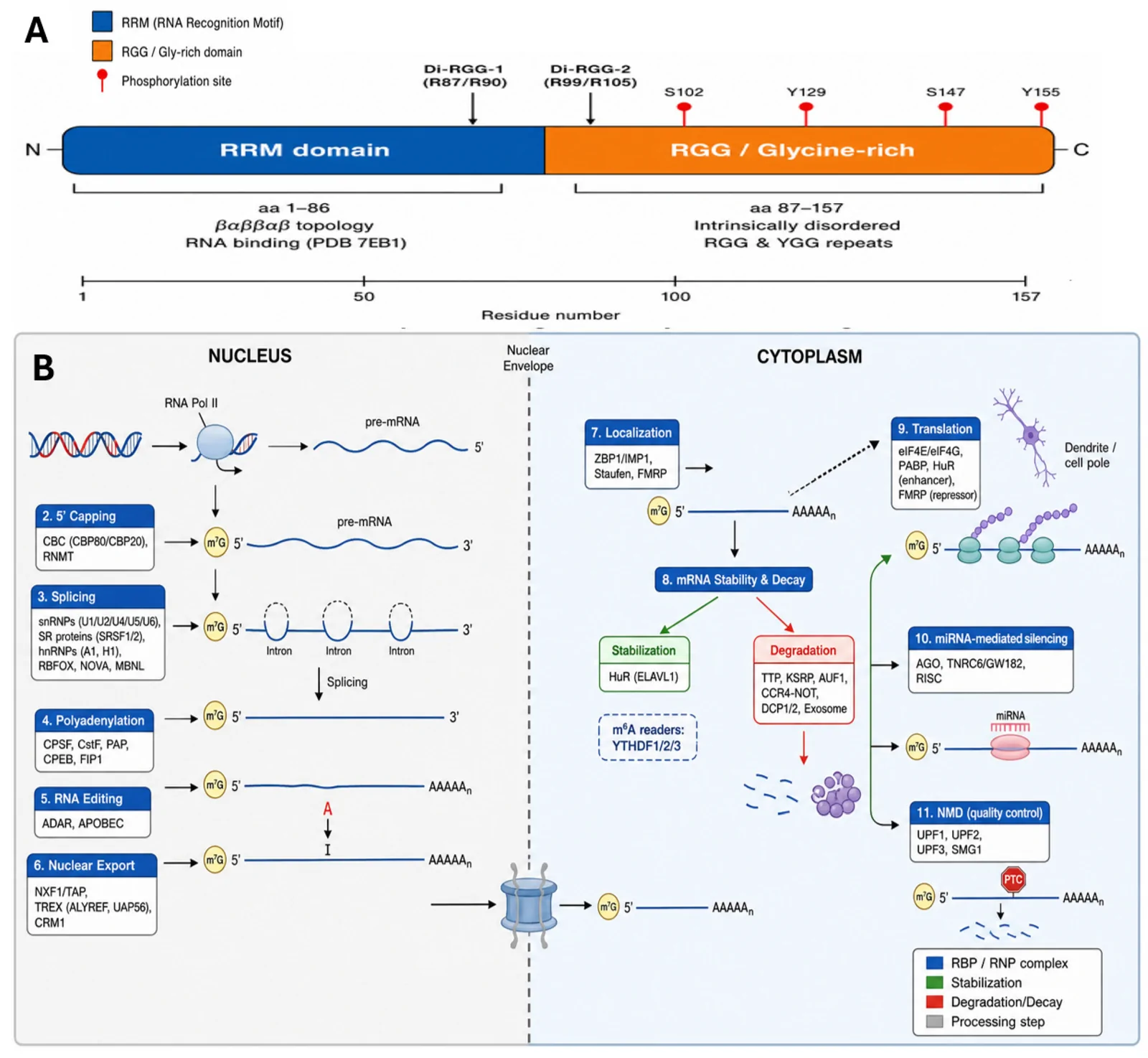
The regulation of gene expression after transcription, influenced by RNA-binding proteins. (**A**). The RRM domain and RGG domain of RBM3 are shown. (**B**). The posttranscriptional regulation is controlled by RNA-binding proteins.

RNA-binding proteins (RBPs), as their name implies, represent a category of proteins that create complexes with various nucleic acids, including microRNAs, messenger RNAs, small inducible RNAs, small nuclear RNAs, small nucleolar RNAs, transfer RNAs, and noncoding RNAs [1]. Moreover, they may sporadically bind to single-stranded DNA [2]. It is estimated that 7.5% of the human protein-coding genome comprises RBPs [1]. As shown in Figure 1, these proteins are crucial in numerous cellular processes such as pre-mRNA splicing, editing, polyadenylation, localization, turnover, translation, and degradation [3]. Due to their significant roles in cellular functions, RBPs are subject to strict regulation, and any dysregulation, mutations, or mis-localization can lead to various diseases. RBPs contribute to various diseases due to mutations that can lead either to changes in their expression levels or expression of RBPs with altered functionality. Gebauer et al. demonstrated via gene ontology analysis that mutated RBPs are predominantly associated with metabolism and nervous system development [4]. In fact, they show that RPBs are the most affected class of proteins outranking transcription factors. Interestingly, in cancers the trend is reversed. Moreover, the role of RBPs in cancers appears to be context-dependent. For example, RBM10 somatic mutations have been described in multiple cancers, sometimes leading either to tumor suppression or tumor promotion [5,6,7]. Gebauer et al. also note that currently there is little understanding of mechanistic consequences of these mutations and their contributions to the disease states. Notably, RBPs like TDP-43, Matrin-3, and FUS are suspected to be associated with amyotrophic lateral sclerosis (ALS), a severe neurodegenerative disorder [8,9]. Additionally, RBPs have been implicated in Alzheimer’s disease, Parkinson’s disease, different types of cancer, and cardiovascular conditions [10,11,12,13]. Recently, RBPs have emerged as promising targets for therapeutic interventions, sparking renewed interest in uncovering the underlying molecular mechanisms governing their functions [14].

RNA-binding proteins engage with nucleic acids through one or more domains. Various types of domains are responsible for recognizing RNA, such as the RNA-binding domain (RBD), also referred to as the ribonucleoprotein (RNP) domain or the RNA recognition motif (RRM), the double-stranded RNA-binding domain (dsRBD), the K-homology (KH) domain (type I and type II), the zinc finger (ZnF, predominantly C-x8-X-x5-X-x3-H), the Sm domain, the DEAD/DEAH box, the cold-shock domain, the Pumilio/FBF (PUF or Pum-HD) domain, and the Piwi/Argonaute/Zwille (PAZ) domain [1]. A single domain of this kind has the capacity to bind 2–6 nucleotides. The presence of multiple copies of these domains enhances the binding of more intricate nucleotide sequences and contributes to the specificity of the sequence. The RRM domain is notably prevalent in more complex organisms [15]. Alongside these structured domains, glycine–arginine-rich (GAR/RGG/RG/GRG) motifs are frequently identified as supplementary regions in numerous human RBPs. These motifs are inherently disordered, have an affinity for RNA, and have been associated with various disorders, including neurodegenerative and neuromuscular conditions, as well as cancer [16,17,18].

However, there are also RBPs that lack conventional RNA-binding domains [19]. For example, cyclins A2, L1 and T1 were identified in the nuclear RNA-binding proteome of mouse embryonic stem cells [20]. Mice with reduced cyclin A2 expression are prone to tumor formation and chromosomal instability due to a predisposition to form lagging chromosomes and chromatin bridges [21].

The RNA-binding motif protein 3 (RBM3) is part of the glycine–arginine (GR)-rich RBP family protein, characterized by having one N-terminal RNA-binding domain (RRM) and a highly flexible C-terminal GR-rich region [22]. Due to the presence of a single RRM domain, RBM3 falls within the class IVa-GRP subfamily, whose constituents may harbor up to three RRM domains. RBM3 gene is coded on the X chromosome [21], and its upregulation has been documented in response to cold stress conditions [23].

Utilizing cDNA selection with a YAC originating from the Xp11.2 locus, a novel gene (RBM3) has been discovered, which codes for a polypeptide exhibiting significant sequence homology to a cluster of proteins known to interact with RNA. Positioned on a YAC contig map, RBM3 is situated amidst OATL1 and GATA1/TFE3 in sub-band Xp11.23 and generates alternatively spliced transcripts across various human tissues. The primary open reading frame encompasses a 157 amino acid protein with an anticipated molecular weight of 17 kDa. The presumed RNA-binding domain of RBM3 bears strong resemblance to that of two previously identified human RNA-binding proteins—YRRM, associated with azoospermia, and hnRNP G, a glycoprotein recognized as an autoantigen. The likeness of RBM3 to an RNA binding protein from maize, AAIP, in terms of both sequence and size, indicates its potential involvement in a fundamental pathway conserved from plants to mammals [24,25]. Within the realm of cellular processes, RBM3 exerts significant influence on the foundational translational apparatus, impacting overall protein synthesis, cell proliferation, and functioning as a regulator of programmed cell death. Moreover, RBM3 is associated with tumorigenesis, cancer spread, and neuroprotection [21,25].

This review summarizes the current knowledge of RNA-binding motif protein 3 (RBM3), including its molecular functions, role in disease pathogenesis, prognostic significance, and therapeutic potential. The literature surveyed spans from the initial characterization of RBM3 in 1995 through December 2025, with particular emphasis on studies published between 2010 and 2025 that investigated its biological functions, involvement in cancer and neurodegenerative diseases, and emerging clinical applications.

To provide an overview of the context-dependent biological functions and clinical significance of RBM3, Table 1 summarizes its expression patterns, molecular mechanisms, experimental evidence, and therapeutic implications across major disease settings.

## 2. Importance of RBM3 in Disease Progression

### 2.1. Molecular Functions of RBM3 in Disease Pathogenesis

As stated earlier, RBM3 belongs to a small family of cold-inducible RNA-binding proteins and is involved in regulating various aspects of mRNA metabolism and possesses pleiotropic functions in cell stress, development, and oncogenesis [23,24]. It has been shown that at a molecular level, RBM3 facilitates global protein synthesis, enhances the stability of mRNAs containing AU-rich elements, and aids in the production of numerous microRNAs during the Dicer step [40]. These functions collectively suggest that RBM3 exerts a wide-ranging and distinct regulatory impact on the proteome. At a cellular level, initial investigations have suggested that RBM3 plays a crucial role in adaptive responses to hypothermia, potentially functioning as an mRNA chaperone that preserves translation capability until euthermic conditions are restored [41,42,43,44]. However, it has become evident that RBM3 is triggered by a diverse array of physiological stresses, including hypoxia, endoplasmic reticulum (ER) stress, excitotoxins, radiation, wasting, and certain pathological conditions [21,45,46]. Within these various scenarios, RBM3 exerts a potent cytoprotective effect. Within muscle cells, the induction of RBM3 opposes necrosis and apoptosis, and maintains cellular structure in the face of reactive oxygen species and conditions leading to cellular atrophy [47]. In neuronal cells, RBM3 serves as a shield against cell death brought on by endoplasmic reticulum (ER) stress, hypoxia/ischemia, and a range of other metabolic and disease-related stresses [21,28,45,48,49,50,51,52,53]. These findings suggest a broad function of RBM3 in cell protection. Supporting this idea, RBM3 is the sole transcript that is upregulated across all tissues during torpor, a condition characterized by prolonged hypothermia that spares many tissues, including muscle and brain, from the adverse effects of severe metabolic stress [54]. Apart from being influenced by physiological stressors, the expression of RBM3 displays variations in different brain regions and stages of development and has been implicated in oncogenesis as a potential proto-oncogene and a predictor of the clinical outcome in various cancers [55,56].

Multiple observations indicate that RBM3 possesses morpho-regulatory functions that are pertinent to its involvement in cell protection and significant contribution to both development and oncogenesis [57]. The protein RBM3 (P98179, RNPL) was included in the group of RNA-binding proteins that were found in the invasive pseudopodia of mesenchymal breast cancer cells. Additionally, RBM3 was present in specialized cellular regions known as spreading initiation centers (SICs) that emerge during the initial phases of cell spreading in fibroblasts and various mesenchymal cell lines [57,58,59]. Various categories of RNA-binding proteins (RNA-BPs) have been recognized within SICs and other cellular extensions, such as podosomes, pseudopodia, filopodia, growth cones, and dendritic spines [60,61,62,63,64]. The functions performed at these specific locations are diverse, encompassing the regulation of mRNA positioning and translation processes that facilitate cellular adhesion, expansion, movement, and the direction of projections, along with interactions between proteins that control the dynamic nature of the cytoskeleton. Considering this information, the presence of RBM3 within stress-induced cytoplasmic inclusions (SICs) and invading filopodia implies its involvement in shaping cell morphology and movement, potentially crucial for its stress response mechanisms and speculated role in both embryonic development and oncogenesis [58,59,64,65,66,67]. Studies have shown that the induction of RBM3 provides synaptic protection in experimental models of prion and Alzheimer’s-related neurodegeneration. RBM3 has also been implicated in maintaining neuronal integrity and synaptic homeostasis, as discussed in the neuroprotection section below. Conversely, the depletion of RBM3 worsens the loss of synapses and the inadequacies in synapse regeneration in these models [41,68]. This protective effect of RBM3 on synapses, which might be linked mechanistically to the capacity for synaptic regeneration observed during torpor and cold shock, lends support to the notion that RBM3 may play a role in controlling the development and adaptability of cellular projections. Collectively, these data imply that RBM3 plays critical roles in the regulation of cellular morphology and migration, although this aspect has not yet been fully clarified [69,70].

J. Pilotte et al. provided evidence that RBM3 functions as a controller of cell spreading, polarity, and migration [71]. They also observed that RBM3 is present in various types of cell protrusions found in different cell lines and primary myoblasts. Altering RBM3 expression leads to significant modifications in cell polarity and spreading through the involvement of the RhoA-Rho associated protein kinase (ROCK) signaling pathway and the collapsin response mediator protein 2 (CRMP2). Their findings suggested that these effects may be linked to mechanisms that regulate cell motility. Also, they indicated that RBM3 facilitates directed cell migration in different scenarios by adopting a mesenchymal-like mode characterized by the extension of elongated protrusions. Conversely, reduced levels of RBM3 hinder migration and the ability of cells to elongate protrusions, prompting a shift towards a migration mode reminiscent of amoeboid motion [71].

### 2.2. Importance of RBM3 in Neuroprotection

**Figure 2:**
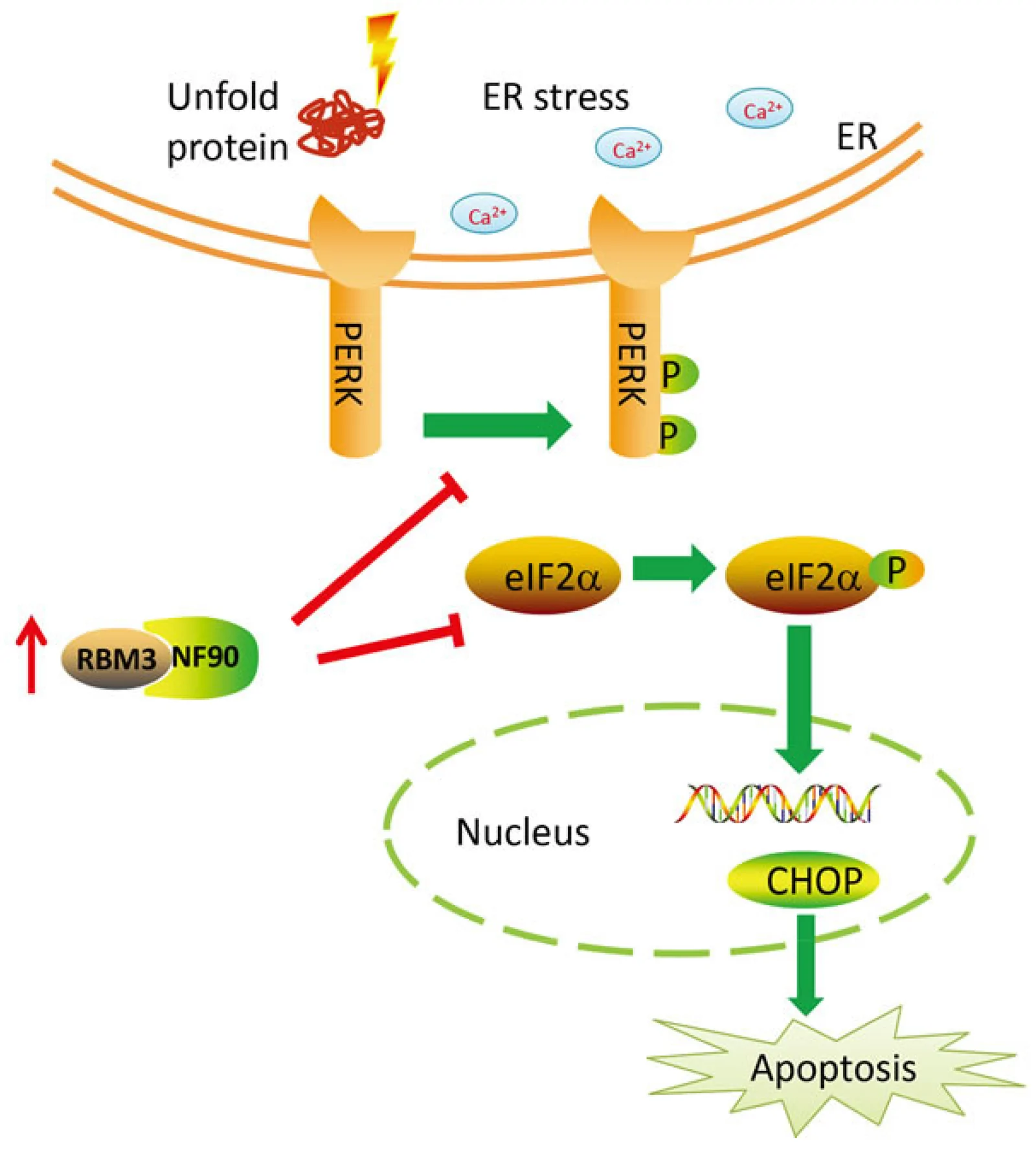
A model illustrating how RBM3 provides neuroprotection by suppressing the ER stress pathway involving PERK, eIF2α, and CHOP. RBM3 functions as an inhibitor of PERK through a mechanism that depends on NF90. By blocking the PERK-eIF2α-CHOP pathway involved in ER stress, RBM3 helps prevent cell apoptosis. Reprinted from ref. [55].

The upregulation of cold-inducible RNA-binding protein (CIRP) via mild hypothermia results in the inhibition of the mitochondrial apoptosis pathway. Consequently, this leads to a reduction in neuronal apoptosis, resulting in safeguarding the neurons from degeneration and subsequent apoptosis. These results validate the advantageous effects of employing mild hypothermia therapy for brain injury [72]. Beyond its role as a cold-shock protein, RBM3 has emerged as an important regulator of cellular stress responses, neuronal survival, and tumor biology. Recently, researchers have demonstrated the involvement of RBM3 in facilitating neuroprotection induced by hypothermia. It has been shown that a high level of RBM3 expression is associated with the development of the neurons of the murine brain. Moreover, the enhancement of the expression of RBM3 has been linked to a decrease in the degree of neuronal cell death. Furthermore, the protective impact of hypothermia has been compromised as a result of downregulation of RBM3 expression. Again, studies have demonstrated that neuronal cells acquire greater resistance to cell death by stimulating RBM3 even under normal body temperature conditions (37 °C) [50]. Similarly, another group reported that RBM3 provides neuroprotection by suppressing the ER stress pathway involving PERK, eIF2α, and CHOP as shown in Figure 2. Ávila-Gómez et al. demonstrated that RBM3 promotes neuroprotection and repair in stroke models by reducing apoptosis, enhancing neurogenesis, and modulating pathways like RTN3 and Nrf2 suggesting that hypothermia increases RBM3 levels, correlating with better outcomes [27].

In contrast, downregulation of RBM3 was linked to synaptic degeneration, negating the neuroprotective benefits of hypothermia and hastening disease progression. Overall, a potential shielding role associated with enhancing cold-stress pathways in neurodegenerative conditions was identified [73]. Furthermore, another study indicated that RBM3 modulates cell proliferation and apoptosis following spinal cord trauma [28,74]. The neuroprotective effect of hypothermia may involve several pathophysiological mechanisms, including an enhancement in protein misfolding and denaturation, a decrease in cell cycle progression, a suppression of translation and transcription processes, a perturbation in cellular cytoskeletal components, and changes in membrane permeability. These alterations ultimately result in elevated cytosolic levels of Na^+^ and H^+^ ions [75]. Studies have consistently demonstrated that mild-to-moderate hypothermia induces RBM3 expression, contributing substantially to its neuroprotective effects. The upregulation of RNA-binding motif protein 3 (RMB3) is particularly evident in specific regions such as juvenile and mature neurons under conditions of lower temperatures [76]. The neuroprotective efficacy of hypothermia has waned due to the suppression of RBM3 expression. However, upregulation of RBM3 has been shown to mitigate the cleavage of poly ADP ribose polymerase (PARP), prevent transnucleosomal DNA fragmentation, and inhibit the release of lactic acid dehydrogenase (LDH) in the absence of hypothermia. These findings are consistent with a recent study indicating the crucial involvement of RBM3 in the neuroprotective mechanisms induced by hypothermia [50]. RBM3 is important in facilitating translation within neuronal cells. Moreover, RBM3 plays a crucial role in providing neuroprotection in primary neurons, PC12 cells (a type of catecholamine cell that synthesize, store and release norepinephrine and dopamine), and cortical organotypic slice cultures when exposed to hypothermic conditions [76].

The therapeutic relevance of RBM3 is not restricted to prion disease and Alzheimer’s disease; emerging data suggests broader neuroprotective roles across multiple CNS pathologies. Following ischemic stroke, experimental induction of RBM3 attenuates apoptosis, preserves synapses, and drives neuronal survival. These multifaceted mechanism-driven benefits highlight RBM3 modulation as a compelling strategy for accelerating neurological recovery post-stroke [27]. Following traumatic spinal cord injury, RBM3 exhibits dynamic spatiotemporal regulation. Evidence suggests this upregulation serves a neuroprotective role, promoting cell survival and facilitating neuronal repair mechanisms under cellular stress [28,48]. In neurodegenerative conditions like Parkinson’s disease and ALS (amyotrophic lateral sclerosis), aberrant RNA-binding protein activity represents a key pathogenic driver. While the specific contribution of RBM3 to these diseases is not yet completely understood, its established functions—namely, maintaining protein homeostasis, modulating cellular stress, and preserving synaptic integrity—suggest that targeting RBM3 could offer significant therapeutic potential [26,52,77,78].

Collectively, these findings support continued investigation of RBM3 as a potential therapeutic target for a broad range of CNS diseases characterized by synaptic dysfunction, progressive neuronal loss, and impaired cellular stress responses.

### 2.3. The Expression of RBM3 in Cancer

**Figure 3:**
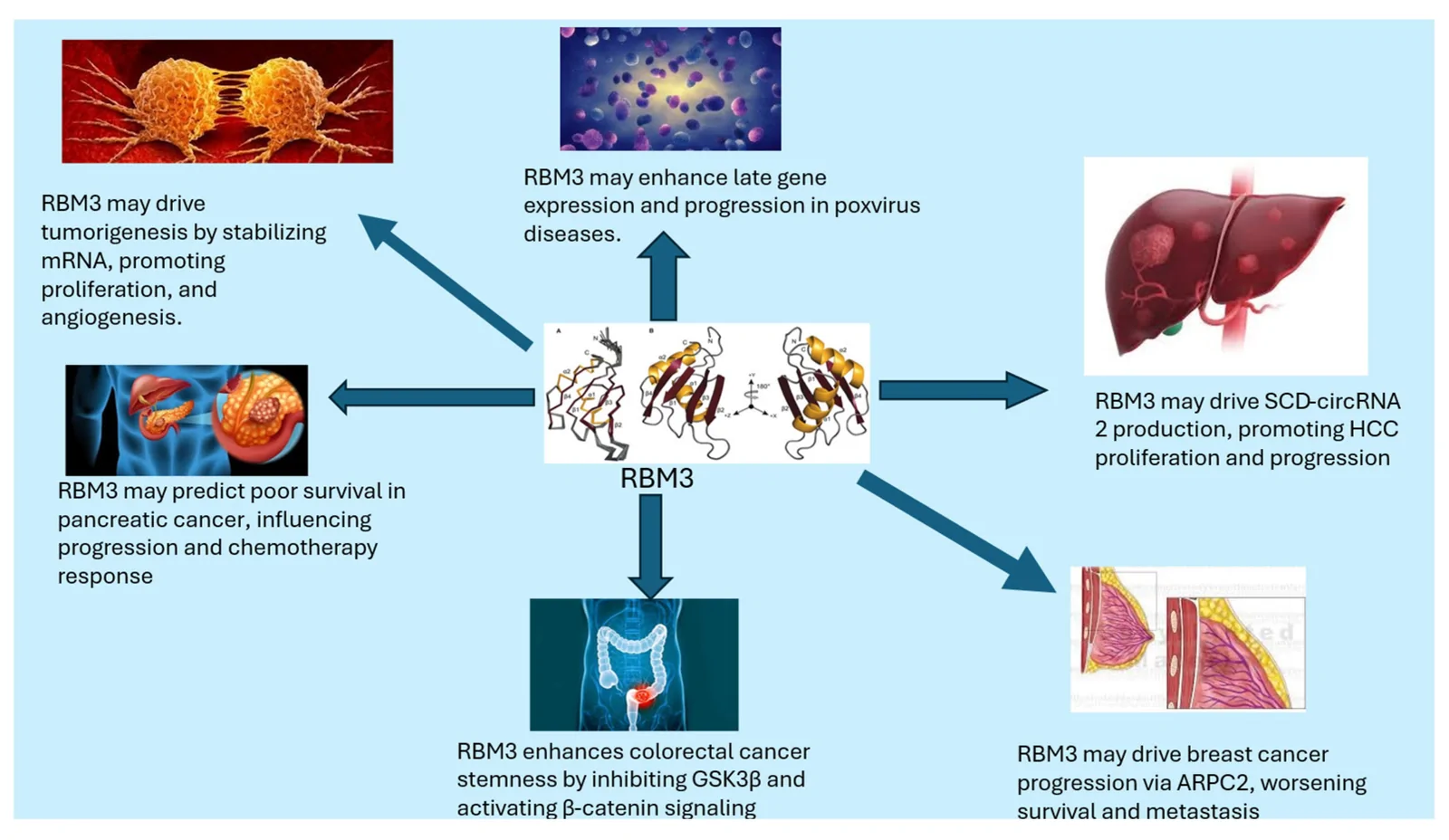
Role of RBM3 in tumorigenesis. RBM3 plays a pivotal role in tumorigenesis across multiple cancers by modulating molecular pathways and influencing cellular processes. It stabilizes mRNA of tumorigenic factors (COX-2, IL-8, VEGF) and drives proliferation, angiogenesis, and metastasis in HCC, breast, pancreatic, and colorectal cancers. Additionally, RBM3 enhances β-catenin signaling, interacts with YAP1, and regulates apoptosis, suggesting its potential as a prognostic marker and therapeutic target [55].

RNA-binding proteins (RBPs) are responsible for governing multiple stages of protein synthesis and the regulation of post-transcriptional gene expression; therefore, they represent an important area of focus within the field of cancer research. RBPs have been demonstrated to fulfill a significant role in RNA metabolism by overseeing processes such as RNA splicing, transportation, monitoring, degradation, and translation. Disruptions in the expression of RBPs have been shown to impact the various stages of RNA metabolism, leading to altered RBP expression and malfunction in the pathogenesis of diverse diseases [68]. The development of cancer and neurodegenerative disorders can be attributed to disruptions in the process of splicing, particularly alternative splicing, which can be linked to the production of cell surface proteins. According to Kim et al. (2009), these alterations in splicing patterns may serve as potential predictor of therapeutic response for both diagnosing and categorizing cancer [68]. Also, Kechavarzi and Janga (2014) performed a comparative analysis of gene expression profiles in cancerous and healthy tissues, revealing a notable upregulation of approximately 30 RNA-binding proteins (RBPs) in at least six out of the nine examined cancer cases [79]. Many studies have been conducted to examine the significance of RBM-3 in various human tissue types. RBM3 has been identified as a potential indicator for predicting treatment outcomes and prognosis in different forms of human cancer, such as prostate, breast, and ovarian cancer [29] and as shown in Figure 3. A study conducted by Jogi et al. (2009) used an antibody-based proteomics strategy to examine the levels of RBM3 protein in both tumor and normal tissue samples [29]. Based on immunohistochemistry and tissue microarrays in two distinct breast cancer patient groups, it was reported that nuclear RBM3 expression was linked to smaller, lower-grade, and ER-positive tumors. Also, in both patient cohorts, the upregulation of nuclear RBM3 protein expression was associated with improved overall survival and a lower risk of recurrence. It was also observed that elevated levels of nuclear RBM3 expression were especially prominent in hormone receptor-positive tumors, as confirmed by multivariate analysis involving tamoxifen treatment controls [29].

RBM3 has been found to serve as a predictor of therapeutic response in epithelial ovarian cancer patient cohorts. The expression of RBM3 has been examined in vitro using both cisplatin-sensitive and cisplatin-resistant ovarian cancer cell lines. An increased expression of RBM3 at both the protein and mRNA levels were observed in the cisplatin-sensitive cells as compared to their resistant counterparts. Silencing of RBM3 expression via small interfering RNA (siRNA) led to an enhanced cisplatin resistance, as evidenced by increased cell viability and a higher proportion of cells arrested in the G2/M-phase [30].

Another expression study of RBM3 in epithelial ovarian cancer using tissue microarrays obtained from 183 patients demonstrated high expression of RBM3 in 51.4% of cases. High expression of RBM3 correlated with favorable histologic subtypes (mucinous, endometrioid, clear cell), early International Federation of Gynecology and Obstetrics (FIGO) stage, and the absence of distant metastasis. Despite this correlation with favorable histologic subtypes, there was no significant difference in survival based on RBM3 expression. Based on these results, the authors suggested that RBM3 may serve as a complementary biomarker for risk stratification and prognosis in clinical practice [32].

In addition, studies have been conducted on epithelial ovarian carcinoma to explore the molecular mechanisms underlying the prognostic significance of RBM3 in epithelial ovarian cancer (EOC). Gene analysis showed a connection between RBM3 expression and various cellular processes linked to the preservation of DNA integrity, including DNA replication, chromatin remodeling, and DNA integrity checkpoint mechanisms. Findings indicate a relationship between unfavorable prognosis in EOC patients and elevated levels of checkpoint kinase 1 and 2 homologues (Chk1 and Chk2) proteins. Thus, RBM3 may participate in cellular responses to DNA damage, potentially elucidating its cisplatin-sensitizing attributes and favorable prognostic impact in EOC. Moreover, studies have shown that Chk1 and Chk2 serve as indicators of poor prognosis and treatment response prediction in EOC [31].

In malignant melanoma, the expression of minichromosome maintenance 3 (MCM3) has been identified as a predictor of therapeutic response, indicating a poor prognosis. An inverse relationship between RBM3 expression and MCM3 expression has been reported in previous studies [80,81]. These findings are similar to the conclusions drawn by Ehlén et al. (2011), who suggested that MCM3 expression is associated with unfavorable outcomes and has potential clinical use as a prognostic indicator in EOC [31]. Experimental data obtained from an in vitro study revealed a notable decrease in RBM3 gene expression as melanoma cells proliferated. Consistent with the above findings, it has been established that RBM3 expression is reduced in metastatic melanoma compared to primary tumors, where strong nuclear RBM3 expression is correlated with positive clinicopathological characteristics and extended overall survival [82].

Immunohistochemistry studies were conducted on colorectal cancer (CRC) cells to assess the prognostic significance of RBM3 in two distinct patient cohorts’ tumors, with the use of both monoclonal and polyclonal antibodies. The results showed that the nuclear expression of RBM3 was linked to enhanced overall survival in CRC patients, along with a strong correlation between the two types of antibodies [36]. In colorectal tumors, elevated levels of nuclear and cytoplasmic RBM3 expression were noted. The regulatory mechanism of RBM3 was elucidated through its binding to AU-rich sequences in the 3’UTR of cyclooxygenase-2 (COX-2). Overexpression of RBM3 induced anchorage-independent growth in non-transformed cells and increased cell proliferation, while the depletion of RBM3 resulted in decreased proliferation of colon adenocarcinoma cells [36].

Another group found that RBM3 overexpression in prostate cancer cells led to a reduction in their tumorigenic potential in vivo and their stem cell-like properties in vitro [83]. It has also been noted that the splicing of RNA in the CD44 variant v8-v10 was inhibited either by overexpressing RBM3 or by culturing cells at 32 °C, resulting in an elevation in the expression of the standard CD44 protein (CD44s) isoform. Conversely, the ratio of CD44v8-v10 to CD44s mRNA was increased by silencing RBM3 or by culturing cells in soft agar. Mechanistic analyses showed that the promotion of CD44v8-v10 hindered the expression of cyclin D1 and coincided with MMP9-mediated cleavage of CD44s. Meanwhile, the siRNA-mediated suppression of CD44v8-v10 reduced the capacity of prostate cancer cells to form colonies in soft agar. Additionally, RBM3 played a role in the stem cell-like characteristics of prostate cancer by inhibiting the splicing of CD44v8-v1096. Moreover, Jonsson et al. (2011) reported that RBM3 was significantly upregulated in various fractions and intensities in invasive carcinoma and prostatic intraepithelial neoplasia [34]. In contrast, RBM3 was seldom expressed in normal prostatic gland epithelium. Thus, the immunohistochemical evaluation of RBM3 expression may serve as an important tool for the stratification and prognostication of prostate cancer patients. Moreover, Boman et al. (2013) suggested that the assessment of RBM3 expression in samples obtained from urothelial bladder cancer could offer greater value for the early diagnosis, staging, prognosis, stratification, and monitoring of disease progression [84].

A study was conducted by Jonsson et al. to assess the clinicopathological associations and prognostic implications of RBM3 expression in tumors originating from a consecutive series of upper gastrointestinal adenocarcinomas. The findings indicated that reduced RBM3 expression is considered an independent prognostic factor for unfavorable outcomes in individuals diagnosed with adenocarcinoma affecting the upper gastrointestinal tract [85]. Also, RBM3 expression levels were found to be elevated in tumors displaying gastric intestinal metaplasia or Barrett’s esophagus as compared to those lacking precursor lesions. These findings reveal a potential role of RBM3 in diverse pathways contributing to the development of esophagogastric junction, esophageal, and gastric adenocarcinomas [85].

Collectively, these findings support RBM3 as a promising prognostic and treatment-response biomarker across multiple malignancies.

Although RBM3 expression has been extensively studied in multiple malignancies, its clinical significance varies according to tumor type, subcellular localization, and treatment context. In most cancer types, elevated nuclear RBM3 expression is associated with favorable clinical outcomes; however, conflicting findings have been reported in certain malignancies, suggesting context-dependent biological functions. Furthermore, RBM3 has been investigated as both a prognostic biomarker, reflecting disease outcome, and a predictive biomarker, indicating the likelihood of treatment response. Prognostic biomarkers provide information regarding disease outcome independent of treatment, whereas predictive biomarkers identify patients who are likely to benefit from a specific therapeutic intervention. To facilitate comparison across studies, the major clinical findings involving RBM3 in human cancers are summarized in Table 2.

## 3. Preclinical and Clinical Studies About RBM3

As discussed earlier, RBM3 has potential clinical use. A study conducted by Wahlin et al. made an argument that RBM3 may serve as a marker of chemotherapy sensitivity and treatment benefit in muscle-invasive bladder cancer. They highlighted the importance of RBM3 as a predictive biomarker and presented clinical data suggesting the importance of neoadjuvant chemotherapy in MIBC (muscle-invasive bladder cancer) patients with high nuclear RBM3 levels. Overall, high levels of RBM3 in neoplastic cells were associated with enhanced survival rates among individuals undergoing neoadjuvant chemotherapy. Also, they found out that suppression of RBM3 in malignant bladder carcinoma cells of high grade was shown to diminish their susceptibility to cisplatin and gemcitabine. Moreover, they reported that the capacity of RBM3 to serve as a prognostic indicator for the reaction to chemotherapy in muscle-invasive bladder cancer is worth considering [38]. Also, there has been an association between reduced expression of RBM3 and advanced tumor stage in non-small cell lung cancers (NSCLCs), especially in cases of lung adenocarcinomas (LUACs). The diminished presence of RBM3 is indicative of a poor prognosis in LUAC patients, correlating with a shortened overall survival [86]. Also, it was proposed that RBM3 may function as an independent predictor for overall survival in individuals diagnosed with primary malignant melanoma [82]. Clinical studies consistently demonstrate that high nuclear RBM3 expression is associated with favorable prognosis in breast cancer, particularly in ER-positive disease.

Similarly, it was found that enhanced nuclear RBM3 expression correlates with enhanced overall and recurrence-free survival rates in breast cancer, and RBM3 may serve as an independent predictor of favorable clinical outcome in breast cancer, particularly in ER-positive tumors [29]. Clinical observations further support the association between elevated RBM3 expression and favorable outcomes in prostate cancer; thus, RBM3 may serve as a potential biomarker for a favorable prognosis of the disease. This cohort analysis was done on 88 patients treated with radical prostatectomy for localized disease [34].

## 4. Discussion

Zhou et al. admit that in cancer, the expression level and clinical behavior of RBM3 is very conflicting; thus, further studies of the regulatory mechanisms of RBM3 in oncogenic or tumor suppression are greatly needed both in vitro and in vivo. As a potential biomarker, there is a need for further studies to evaluate the correlation between the expression level of RBM3 in diverse cancer stages to categorize patients for individualized prevention and treatment. In neuroprotection, though it has been shown that RBM3 is a promising therapeutic candidate for neuroprotection in prion-infected and 5XFAD models, it must be done in humans before drugs are developed to mimic the protective effects of cooling [55]. Also, additional experimental evidence is needed to establish the importance of RBM3 as a promising target in cancer diagnosis and therapy, investigating the molecular mechanisms that promote the expression of RBM3 under adverse conditions and also in cancer [89]. Again, there is a need for more comprehensive studies for alternative explanations for the observed effects of RBM3 on tumor cells and immune cells in the tumor microenvironment. The mechanism of action of RBM3 in certain diseases, including cancer, particularly in prostate cancer, is not well-known; therefore, further investigations are needed.

Research has shown that the upregulation of RBM3 may be linked to certain diseases such as poxvirus disease [90], hepatocellular carcinoma [39], breast cancer [91], pancreatic [88] and colorectal cancer [37]. However, there is not enough evidence to prove this. Since this is a potential concern, we need more experimental and clinical data to prove or disprove this concept. This will help us to know if we can limit toxicity and achieve a good safety profile in case there are molecules that are being discovered primarily to upregulate RBM3 to treat various conditions, including cancer.

One of the approaches that can help define direct RBM3 RNA targets in disease-relevant models is transcriptome-wide binding maps described in [92]. This comprehensive approach involves (1) detection of in vivo binding activity using enhanced CLIP (eCLIP) assays, (2) defining the in vitro binding specificities of using RNA Bind-N-Seq assays and identifying the connections between in vitro and in vivo binding, (3) mapping the subcellular localization of RBPs using immunofluorescence, to understand widespread organelle-specific regulation of RNA processing, and (4) profiling the DNA association patterns RBPs by chromatin IP and sequencing (ChIP–seq), to define broad interconnectivity between chromatin association and RNA processing.

An important question for future drug discovery efforts is identifying the disease setting in which RBM3 modulation is most likely to provide therapeutic benefit. Among the malignancies reviewed, prostate cancer appears to be one of the most promising candidates for RBM3-targeted therapeutic development. Experimental studies have demonstrated that RBM3 suppresses stem-like properties, reduces tumorigenic potential, and modulates CD44 variant splicing in prostate cancer cells [83]. Furthermore, elevated RBM3 expression has been associated with a reduced risk of biochemical recurrence and disease progression in patients with prostate cancer [34]. Given the substantial clinical burden of this disease, the therapeutic potential of RBM3 warrants further investigation. According to the American Cancer Society, an estimated 333,830 new cases of prostate cancer and 36,320 prostate cancer-related deaths are expected in the United States in 2026. Together, the biological evidence and disease burden support prostate cancer as a particularly attractive indication for the development of RBM3-based therapeutic strategies. Future studies should focus on elucidating the molecular mechanisms underlying RBM3-mediated tumor suppression and determining whether pharmacological induction of RBM3 can be translated into clinically meaningful therapeutic benefit.

So far, most of the experiments done show a good safety profile when RBM3 is upregulated in preclinical trials [27,33,38,39,55,83,93].

Although RBM3 has emerged as a promising therapeutic target, a search conducted as of July 2026 on open targets platform shows that there is no approved drug currently listing RBM3 as a direct molecular target or mechanism of action [94]. Nevertheless, some compounds have been reported to modulate RBM3 expression in preclinical studies. Among RBM3-inducing agents, neurotoxin domoic acid, fibroblast growth factor 21 (FGF21), melatonin, and the synthetic compound ZR17-2 have been shown to increase RBM3 expression in rodent models and in vitro systems [46,48,95,96]. Conversely, metformin and the AMP analog AICAR (5-aminoimidazole-4-carboxamide-1-β-D-ribofuranoside, acadesine) have been reported to suppress RBM3 expression in vivo and in vitro through activation of AMP-activated protein kinase (AMPK)-dependent signaling pathways that influence alternative splicing [97]. These findings demonstrate that RBM3 can be pharmacologically modulated and support the feasibility of developing selective RBM3-targeted therapeutics in the future.

## Figures and Tables

**Table 1:** Summary of RBM3 functions across major disease settings. RBM3 regulates multiple RNA-processing pathways, including mRNA stabilization, microRNA biogenesis, alternative splicing, translational control, and cellular stress responses. These activities contribute to disease-specific effects ranging from neuroprotection and synaptic preservation in central nervous system disorders to context-dependent tumor-promoting or tumor-suppressive functions in cancer.

Disease Context	RBM3 Expression Pattern	Major Molecular Mechanisms	Experimental Model/Evidence	Therapeutic Implication
Alzheimer’s disease	Increased expression is neuroprotective	Synaptic preservation, structural plasticity, suppression of neurodegeneration	Murine neurodegeneration models [26]	RBM3 induction may delay disease progression
Prion disease	Increased expression is protective	Synapse regeneration, reduced neuronal loss	Prion disease models [26]	Potential neuroprotective strategy
Ischemic stroke	Increased expression associated with recovery	Reduced apoptosis, neurogenesis, RTN3 and Nrf2 signaling	Experimental stroke models [27]	Candidate target for neuroprotection
Spinal cord injury	Upregulated after injury	Regulation of apoptosis and cell proliferation	Rat spinal cord injury models [28]	Potential neuroregenerative target
Breast cancer	High nuclear expression associated with favorable outcomes	Regulation of RNA processing and proliferation	Clinical cohorts [29]	Prognostic biomarker
Ovarian cancer	High RBM3 associated with favorable prognosis and cisplatin sensitivity	DNA damage response, Chk1/Chk2 signaling	Patient cohorts and cell lines [30,31,32]	Prognostic and predictive biomarker
Prostate cancer	High RBM3 associated with reduced progression	CD44 alternative splicing, suppression of stem-like properties	Clinical and experimental studies [33,34]	Potential therapeutic target and prognostic biomarker
Colorectal cancer	Context-dependent, may promote proliferation	COX-2 mRNA stabilization, β-catenin signaling	Cell-based and clinical studies [35,36,37]	Requires disease-specific targeting strategy
Bladder cancer	High expression predicts chemotherapy response	Increased cisplatin and gemcitabine sensitivity	Clinical and preclinical studies [38]	Predictive biomarker for neoadjuvant chemotherapy
Hepatocellular carcinoma	Elevated expression associated with tumor progression	Regulation of proliferation pathways	Experimental studies [39]	Possible oncogenic role requiring inhibition

**Table 2:** Clinical significance of RBM3 expression in human cancers. Summary of published clinical studies evaluating RBM3 expression across multiple malignancies. The table distinguishes prognostic and predictive biomarker roles, subcellular localization of RBM3 expression, treatment context, clinical endpoints, and whether RBM3 remained an independent biomarker following multivariable analysis.

Cancer Type	Cohort Size	RBM3 Localization	Prognostic or Predictive Value	Treatment Context	Clinical Endpoint	Independent Biomarker?	Key Reference
Breast cancer	Two independent cohorts	Nuclear	Prognostic	Tamoxifen-treated and untreated patients	Overall survival and recurrence-free survival	Yes	[29]
Epithelial ovarian cancer	267 patients	Nuclear	Prognostic and predictive	Cisplatin-based treatment	Survival and chemotherapy response	Yes	[30]
Prostate cancer	88 patients	Nuclear	Prognostic	Radical prostatectomy	Biochemical recurrence and disease progression	Yes	[34]
Colorectal cancer	Two cohorts	Nuclear and cytoplasmic	Prognostic	Standard management	Overall survival	Yes	[36]
Muscle-invasive bladder cancer	Clinical cohorts	Nuclear	Predictive	Neoadjuvant chemotherapy	Treatment response and survival	Yes	[38]
Non-small cell lung cancer	Clinical cohort	Reduced expression linked to poor outcome	Predictive	Standard care	Overall survival	Yes	[86,87]
Malignant melanoma	215 patients	Nuclear	Prognostic	Standard management	Overall survival	Yes	[82]
Esophageal/Gastric adenocarcinoma	Clinical cohort	Nuclear	Prognostic	Standard management	Recurrence and mortality risk	Yes	[85]
Pancreatic cancer	Multiple cohorts	Nuclear and Cytoplasmic	Prognostic	Standard management	Patient stratification	Uncertain	[88]
Hepatocellular carcinoma	Experimental and clinical studies	Elevated expression	Prognostic	Standard management	Tumor progression	Uncertain	[39]

## Data Availability

No new data were created or analyzed in this study. Data sharing is not applicable to this article.

## Abbreviations

The following abbreviations are used in this manuscript:

RBM3	RNA Binding Motif 3
ALS	Amyotrophic Lateral Sclerosis
CRC	Colorectal Cancer
CRIP	Cold-inducible RNA-binding Protein
EOC	Epithelial Ovarian Cancer
ER	Endoplasmic Reticulum
PARP	Poly ADP Ribose Polymerase
RBD	RNA-Binding Domain
RRM	RNA Recognition Motif
